# Prevalence and predisposing factors for hepatitis C virus in haemodialysis unit Universiti Kebangsaan Malaysia Medical Centre

**DOI:** 10.1186/1753-6561-5-S6-P210

**Published:** 2011-06-29

**Authors:** R Jaafar, I Isahak, KK Wong, Z Zainol Rashid, A Sulong, MH Jaafar, AH Abdul Gafor

**Affiliations:** 1 Department of Medical Microbiology & Immunology , Faculty of Medicine, Universiti Kebangsaan Malaysia Medical Centre (UKMMC), Kuala Lumpur, Malaysia; 2 Faculty of Medicine & Health Sciences, Universiti Sains Islam Malaysia, Kuala Lumpur, Malaysia; 3 Department of Community Health, Kuala Lumpur, Malaysia; 4 Department of Medicine, Faculty of Medicine, Universiti Kebangsaan Malaysia Medical Centre (UKMMC), Kuala Lumpur, Malaysia

## Introduction / objectives

Haemodialysis patients are at risk of infection by hepatitis C virus (HCV), suggestive of nosocomial spread. This study aimed to determine the prevalence and predisposing factors of HCV infection and evidence of outbreak among haemodialysis patients.

## Methods

A cross-sectional study in 3 UKMMC dialysis centres from June 2009 to May 2010 included 35 patients. Data were obtained by review of medical records and interviews with patients and staff. HCV infection was determined by antibody detection using Abbott Axsym® 3.0 and by in-house HCV RNA polymerase chain reaction (PCR). Genotype was determined by sequence analysis and Versant® HCV Genotype 2.0.

## Results

The prevalence of HCV infection was 20.0% with no non-seroconverters. Genotype 1 was predominant (4/7). The predisposing factors were duration of haemodialysis, history of dialysis at outside centres and place of haemodialysis. 3 out of 5 patients with history of dialysis at Mecca seroconverted. Based on epidemiological data, an outbreak with genotype 1 occurred in 2007, involving 3 patients after one of them had haemodialysis at Mecca. Review of laboratory results revealed that 2 of them were first diagnosed by HCV RNA PCR. Sequence analysis was not done in these 3 patients.

## Conclusion

Screening for HCV antibody is inadequate for detection of early HCV infection. HCV RNA PCR is necessary for patients with history of dialysis at outside centres for early infection control measures and prompt treatment.

## Disclosure of interest

None declared.

